# Practice change toward better adherence to evidence-based treatment of early dental decay in the National Dental PBRN

**DOI:** 10.1186/s13012-014-0177-x

**Published:** 2014-12-02

**Authors:** Donald Brad Rindal, Thomas J Flottemesch, Emily U Durand, Olga V Godlevsky, Andrew M Schmidt, Gregg H Gilbert, National Dental PBRN Collaborative Group

**Affiliations:** HealthPartners Institute for Education and Research, 8170 33rd Avenue South, MS 21111R, Bloomington, MN 55425 USA; Dental Clinic Admin, HealthPartners, 8170 33rd Avenue South, MS 21111R, Bloomington, MN 55425 USA; University of Alabama at Birmingham, 1919 7th Avenue South, Birmingham, AL 35294 USA

**Keywords:** Decision-making, Dissemination, Evidence-based medicine, Oral health, Organizational behavior, Performance measurement, Physician practice patterns

## Abstract

**Background:**

Significant national investments have aided the development of practice-based research networks (PBRNs) in both medicine and dentistry. Little evidence has examined the translational impact of these efforts and whether PBRN involvement corresponds to better adoption of best available evidence. This study addresses that gap in knowledge and examines changes in early dental decay among PBRN participants and non-participants with access to the same evidence-based guideline. This study examines the following questions regarding PBRN participation: are practice patterns of providers with PBRN engagement in greater concordance with current evidence? Does provider participation in a PBRNs increase concordance with current evidence? Do providers who participate in PBRN activities disseminate knowledge to their colleagues?

**Methods:**

Logistic regression models adjusting for clustering at the clinic and provider levels compared restoration (dental fillings) rates from 2005–2011 among 35 providers in a large staff model practice. All new codes for early-stage caries (dental decay) and co-occurring caries were identified. Treatment was determined by codes occurring up to 6 months following the date of diagnosis. Provider PBRN engagement was determined by study involvement and meeting attendance.

**Results:**

In 2005, restoration rates were high (79.5%), decreased to 47.6% by 2011 (*p* < .01), and differed by level of PBRN engagement. In 2005, engaged providers were less likely to use restorations compared to the unengaged (73.1% versus 88.2%; *p* < .01). Providers with high PBRN involvement decreased use of restorations by 15.4% from 2005 to 2008 (2005: 73%, 2008: 63%; *p* < .01). Providers with no PBRN involvement decreased use by only 7.5% (2005: 88%, 2008: 82%; *p* = .041). During the latter half of 2008 following the May PBRN meeting, attendees reduced restorations by 7.5%, compared to a 2.4% among non-attendees (OR = .64, *p* < .01).

**Conclusions:**

Based on actual clinical data, PBRN engagement was associated with practice change consistent with current evidence on treatment of early dental decay. The impact of PBRN engagement was most significant for the most-engaged providers and consistent with a spillover effect onto same-clinic providers who were not PBRN-engaged. PBRNs can generate relevant evidence and expedite translation into practice.

**Electronic supplementary material:**

The online version of this article (doi:10.1186/s13012-014-0177-x) contains supplementary material, which is available to authorized users.

## Background

Multiple strategies have been studied to translate current scientific evidence into routine clinical practice, including the development of clinical guidelines that summarize current evidence related to the management of clinical conditions. Nonetheless, having a clinical guideline does not ensure a change in clinical practice [1], and indeed, a review of 59 published evaluations of clinical guidelines concluded that guidelines could improve clinical practice, but the size of the improvements in performance varied considerably [2]. The use of specific strategies to implement research-based recommendations appears necessary to change practice, as more intensive efforts are generally more successful [3].

One means to foster implementation of the latest clinical evidence into routine clinical practice has been practice-based research networks (PBRNs). PBRNs are groups of independent practices focused upon patient care but networked together with the shared goal of learning how care occurs and how outcomes vary across populations and practice settings [4]. By design, PBRNs go beyond a single practice or study and are instead a community-based laboratory encompassing broad patient populations and provider groups. An important part of the mission of a PBRN is to close the *research-to-practice gap*, that is, the gap between what evidence suggests should be occurring in routine practice and what is actually occurring. Gilbert and colleagues used questionnaire data from individual practitioners to provide evidence that dental PBRN involvement can be an effective means to move scientific findings into clinical practice [5,6]. Rhyne and colleagues [7] reported change in physician behavior associated with a PBRN study of acanthosis nigricans, as manifested by increased preventive counseling. This was measured 3–5 years after the study was completed, using a 13-item survey administered by telephone that queried familiarity with the condition before the study, how the study affected clinician behavior, and the value of diagnosis in preventive counseling. Results from chart reviews of study patients led to the conclusion that pediatricians who participate in a PBRN study are more likely to use a study intervention compared to community pediatricians who do not participate in a PBRN. Yawn and colleagues [8] used semi-structured qualitative telephone interviews in a clinical trial of postpartum depression and concluded that PBRN participation provided advantages to practices that extended beyond the study’s specific purpose, such as adaptation of the study tools to other chronic conditions, increased sense of professional self-worth and community recognition, increased research literacy within the practice, and more effective teamwork with staff. Nease and colleagues [9] used qualitative interviews of practitioners and concluded that there had been long-term sustained improvement in depression care. Haines and colleagues [10] reported the first study that combined quantitative and qualitative methods to examine factors that contribute to clinical care networks (not research networks or PBRNs).

Reports that used patient-level data to determine whether PBRN participation fostered adoption of new evidence are limited. Researchers examined the association between Community Clinical Oncology Program (CCOP) affiliation and use of oxaliplatin in community practitioners [11]. Adoption of new evidence was significantly higher among CCOP PBRN members, as judged by whether patients in CCOP practices received oxaliplatin as compared to patients in non-CCOP practices, although the authors could not definitively ascertain that the direct cause was due to CCOP study participation. Bahrami and colleagues utilized clinical records to measure change in practice by comparing different strategies to disseminate a clinical guideline for third molar removal. They did not find a change in practice but the pre-intervention compliance was already high [12], Van der Sanden and colleagues also examined the effectiveness of a third molar guideline implementation strategy that utilized feedback, reminders and an interactive meeting. The intervention was effective compared to a passive control when measuring the outcomes of changes in referral rates and the dentists’ knowledge of the guideline [13]. Mettes [14] and colleagues compared a multifaceted intervention to a passive dissemination about a risk assessment-based guideline that addressed the time interval between oral examination visits and frequency of radiographs for low-risk patients. Their results showed a small to moderate effect on the performance of general dentists. In summary, guidelines are an appropriate strategy for keeping up to date with current knowledge on a topic but will have greater potential to impact patient care if combined with evidence-based multi-faceted strategies and tools to implement the guideline into clinical practice [15]. Botello-Harbaum and colleagues examined the information-seeking behaviors of dental practitioners involved in a three regional dental PBRNs. They found that peer-reviewed sources were more frequently used by full participants [16].

In 2006, the National Dental PBRN [17], formerly called DPBRN [18], began an observational study examining the treatment of previously untreated permanent tooth surfaces. An aim of that study was to measure dentists’ pre-operative and post-operative assessments of the depth of the caries lesion (decayed tooth structure) being treated surgically versus medically (i.e., tooth restoration versus the use of preventive remineralization techniques) to manage the disease process [19]. Another prior study utilizing a questionnaire methodology quantified the depths of caries lesions that lead dentists to intervene restoratively, based on hypothetical scenarios that presented radiographic images and patient background information [20]. The network also held regional meetings of its practitioners to share results from network studies and to discuss potential study topics of interest. The presentation and discussion of results from these two studies was a key dissemination activity during a 2008 network-wide annual meeting of practitioners. Study results were shared along with current literature on the topic of treatment of early caries lesions. The meeting was very participatory, and considerable time was spent discussing strategies to implement the results with colleagues representing diverse geographic regions and practice settings.

The current report focuses upon a natural experiment that occurred within a component of the network called the HealthPartners Dental Group (HPDG). This current study employed a retrospective, observational cohort design examining the treatment of early caries. This study examined the impact of participation in a PBRN on practice patterns associated with the treatment of early caries.

Current evidence [21,22] supports an approach to the treatment of early non-cavitated caries lesions (tooth decay) that incorporates non-invasive remineralization of tooth structure, as compared to a more traditional surgical intervention in which tooth structure is removed and dental restorations (fillings) are placed. This study examined three questions about the impact of PBRN engagement and its impact upon clinical care in the area of early caries treatment. These were as follows:

1. Are the practice patterns of providers (dentists) who participate in a PBRN in greater concordance with current evidence than those who do not participate?
2. Does provider participation in a PBRN increase concordance of current practice with current evidence?
3. Do providers who participate in PBRN activities disseminate knowledge to their colleagues?

## Methods

This study took place at a large, integrated, multi-clinic dental group: HPDG which consists of approximately 60 general dentists and dental specialists practicing in 17 clinic locations located in a large metropolitan area in Minnesota (www.healthpartners.com). This was a natural experiment in that HPDG dentists were encouraged but not required to join either the PBRN (Q1) or—if a PBRN member—attend the 2008 PBRN meeting (Q3). The dentists who joined the PBRN were informed of studies and meetings and could change their level of participation (Q2). This study used observational diagnostic and treatment data gathered from HPDG’s comprehensive electronic dental record (EDR). No data collection protocols were implemented. The caries depth measure is addressed in the HealthPartners caries guideline, and training was provided when the guideline was rolled out to clinics. HPDG’s EDR contains diagnostic and treatment codes on a tooth and surface level. Treatment codes are based upon American Dental Association (ADA) treatment codes.

### Data

This study examined provider practice patterns over time. Key outcomes were identified based on dental restoration treatment codes. Data were obtained from two sources: HPDG’s EDR, which contains diagnostic and treatment codes, and an administrative database that contains provider demographics and tracks their enrollment and engagement within the PBRN.

We identified all HPDG dentists who met the following inclusion criteria: 1) were continuously employed at HPDG from January 1, 2005 through December 31, 2011, 2) actively examined patients for routine visits, and 3) actively performed restorative services for all years during that period.

Within the HPDG system, dental caries and other oral disease are typically diagnosed during a routine examination (performed by dentists) and prophylaxis (cleaning, oral assessment, and preventive services performed by dental hygienists). Any diagnoses by the dentist requiring further care are treatment planned for a subsequent visit. Following this process, we identified these diagnostic codes from HPDG’s EDR: F80 (early stage caries limited to outer half of enamel), F81 (early stage caries extending into inner half of enamel), and F82 (early stage caries extending into outer third of dentin) and classified that examination as the index visit. For each code and tooth, treatment was determined by identifying treatment codes that occurred for a period of up to 6 months following the index visit date.

Treatments were categorically identified (see Additional file 1: Table S1 for codes) as: 1) fluoride, 2) remineralization, 3) restoration, or 4) unidentified with the following order of precedence to determine final treatment for that tooth if co-occurring treatments were identified: restoration, remineralization, and fluoride. This would occur, for instance, if a patient received caries preventive fluoride gel (code 1207) to multiple/all teeth and a tooth with an early caries, lesion was restored during the same or follow-up visit. To allow sharper focus upon our research questions, we then collapsed the treatments to create the final binary outcome of restoration or not.

### Key effect of research question 1

A PBRN study on treatment of early caries was completed in 2007, and initial results were shared through regional meetings and network communications. Dentists were classified based upon their level of PBRN participation: each provider was placed into one of five mutually exclusive levels of PBRN involvement. These levels were the effect of interest for research questions 1 and 2.

1. No PBRN Involvement: did not present research findings at a PBRN meeting, did not collaborate on any PBRN studies, did not respond to PBRN surveys, and did not attend a PBRN meeting
2. Surveys Only: did not present research findings at a PBRN meeting, *responded to 1 or more PBRN surveys*, did not collaborate on any PBRN studies, and did not attend a PBRN meeting
3. Surveys and Studies: did not present research at a PBRN meeting, *responded to 1 or more PBRN surveys, collaborated on 1 or more PBRN studies,* but did not attend a PBRN meeting
4. Surveys, Studies, and Meetings: did not present research at a PBRN meeting, *responded to 1 or more PBRN surveys, collaborated on 1 or more PBRN studies, and attended at least one PBRN meeting*
5. Surveys, Studies, Meetings, and Presentations: *presented research findings at least one PBRN meeting*, *responded to 1 or more PBRN surveys, collaborated on 1 or more PBRN studies, and attended at least one PBRN meeting*

### Key effect of question 2

At the 2008 network-wide PBRN meeting of practitioners, there was a focused dissemination regarding the findings of the PBRN study on the treatment of early caries. The direct impact of these dissemination activities upon meeting attendees was tracked by comparing attendees to non-attendees of this meeting in particular, accounting for this specific measure of PBRN engagement.

### Key effect of question 3

The question of knowledge dissemination across providers was examined by focusing upon non-attendees of the 2008 network-wide meeting. We measured the impact of knowledge dissemination upon restoration rates by focusing upon non-attending providers. We compared restoration rates of those dentists practicing at an HPDG clinic with at least one provider (colleague dentist) who attended the 2008 meeting to providers at HPDG clinics with no providers who attended the 2008 meeting.

### Analysis

The study’s primary outcome was dichotomous: use of restorations in treating early caries lesions compared to an alternative (fluoride, varnish, and/or remineralization). Two multivariable, logistic, mixed-effects regression models adjusting for significant patient and provider factors were used to address our research questions. Models were distinguished by their effect of interest and their analytic sample; however, the empirical approach underlying both was similar.

Each model was developed using a bottom-up approach. First, nesting at the clinic and provider levels was tested. Second, a patient-level model was developed wherein all patient-level factors were screened for significance. Those significant at the 0.10 level were retained. Third, time effects were tested to determine if a secular trend or year-specific effects were preferred. Fourth, provider demographics were screened and incorporated with factors significant at the 0.10 level and/or significantly contributing to model fit (likelihood ratio test ≤ .05) retained. Development of the models and alternative specifications are included in the accompanying appendix.

The first logistic, mixed-effects regression used data from all HPDG providers meeting the three inclusion criteria. The final specification included provider-level random effects and the following patient-level factors: 1) age, 2) number of other enamel findings (the number of other enamel findings diagnosed at the same index visit), 3) number of other dentin findings (the number of other dentin caries diagnosed at the index visit), and 4) number of sealants with damage (the number of teeth previously treated with a sealant that were found to have damage during the visit). This final specification was used to examine research questions 1 and 2. Key variables of interest were provider level of PBRN engagement (Q1) and provider attendance of the 2008 PBRN meeting (Q2).

The second model focused upon research question 3: knowledge dissemination across providers. The data used by that model were limited to those providers who did not attend the 2008 PBRN meeting. This model measured the impact of those dentists who attended the May 2008 meeting upon the practice patterns of those in the provider’s clinic who did not attend by focusing upon practice change among non-attendees within the remainder of 2008.

### Development of analytic dataset

**Figure 1:**
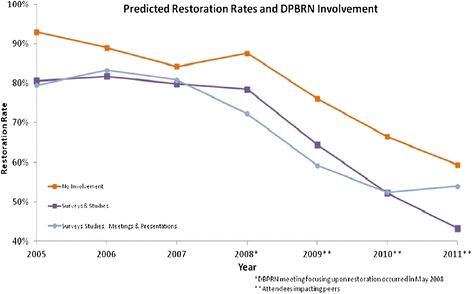
**Restoration rates and network involvement.**

We identified 35 HPDG dentists who met the study’s three inclusion criteria: continuously employed at HPDG from January 1, 2005 through December 31, 2011, actively seeing patients for routine exams, and actively performing restorative services for all years during the period of interest. These were distributed across the five previously described levels of PBRN involvement in the following way: 1) No PBRN Involvement (*n* = 6); 2) Surveys Only (*n* = 4); 3) Surveys and Studies (*n* = 11); 4) Surveys, Studies, and Meetings (*n* = 9); and 5) Surveys, Studies, Meetings, and Presentations (*n* = 5). Our initial analyses included all five levels of PBRN engagement. In subsequent analyses to better distinguish lower and higher levels of PBRN engagement, we combined levels 1 and 2 and levels 3 and 4 (Figure 1) leaving three final levels of PBRN engagement: Low Involvement (levels 1 and 2); Surveys and Studies (levels 3 and 4); and Studies, Meetings, and Presentations (level 5). These levels distinguish cursory engagement (low), those who—at a minimum—attended at PBRN meeting, and those who actively participated and presented PBRN-related research.

From the EDR, we identified 333,959 diagnoses of early-stage carious lesions over the years 2005–2011. We excluded all diagnoses without a clearly identified single provider (*N* = 36,426) or whose identified provider was not among the 35 included in the study (*N* = 100,600).

Table 1 shows the development of the final analytic dataset from these remaining 196,933 diagnoses; 93,698 did not have an identifiable treatment code within the 6-month time window following the index date. This could be for several reasons. First, some of the treatments (e.g., remineralization) are available as over-the-counter rinses and may not have been documented. Second, many adult patients do not have insurance plans that cover caries preventive procedures, such as fluoride or remineralization. Thus, although preventive services were recommended, a patient may have in some cases elected not to treat an asymptomatic presentation, such as an early caries lesion. Third, the dental group is part of several open-access care networks and many patients pursue additional treatment at an alternative provider that is not part of the dental group.

**Table 1:** **Number of findings by study year**

**Total findings**	**2005**	**2006**	**2007**	**2008**	**2009**	**2010**	**2011**	**Total**
By continuously employed dental providers	19,064	21,375	24,384	26,929	30,951	35,981	38,249	196,933
With identified treatment	9,292	10,346	11,691	13,315	16,298	20,235	22,058	103,235
*Percentage*	48.7%	48.4%	47.9%	49.4%	52.7%	56.2%	57.7%	
Identified treatments								
Fluoride	1,687	1,942	2,586	2,946	3,365	4,069	4,947	21,542
Remineralization	206	156	169	490	2,778	5,564	6,620	15,983
Restoration	7,399	8,248	8,936	9,879	10,155	10,602	10,491	65,710
*Restoration rate*	79.6%	79.7%	76.4%	74.2%	62.3%	52.4%	47.6%	
Age distribution of patients								
<18	28.6%	28.7%	26.9%	26.6%	23.5%	21.8%	21.1%	
18–40	44.6%	43.2%	45.9%	46.2%	48.2%	49.2%	49.8%	
40–50	11.1%	11.5%	10.5%	10.9%	11.2%	12.0%	11.7%	
51–65	8.1%	9.8%	9.5%	10.4%	10.8%	10.3%	11.4%	
65–84	6.7%	5.8%	6.1%	4.9%	5.2%	5.4%	4.8%	
85+	0.8%	1.1%	1.1%	1.0%	1.2%	1.3%	1.1%	
Co-occuring findings								
Other enamel diagnostic codes	60.7%	61.3%	60.1%	60.8%	62.9%	61.4%	61.1%	
Mean (sd)	3.9 (4.4)	3.9 (4.2)	3.8 (4.0)	4.0 (4.5)	4.2 (4.4)	4.2 (4.6)	4.2 (4.7)	
Other dentin diagnostic codes	37.9%	39.3%	39.9%	39.4%	40.0%	40.0%	39.1%	
Mean (sd)	2.8 (2.6)	2.8 (2.5)	2.9 (2.7)	2.7 (2.4)	2.6 (2.3)	2.7 (2.4)	2.6 (2.2)	
Repaired sealants with damage	11.0%	11.3%	11.1%	12.4%	9.7%	9.3%	9.4%	
Mean (sd)	3.6 (2.5)	3.5 (2.5)	3.5 (2.2)	3.4 (2.3)	3.3 (2.0)	3.4 (2.2)	3.3 (2.1)	

For the remaining 103,235 findings with a clearly identified treatment, they were classified as a restoration (*n* = 65,710) or non-restorative treatment with the goal of arresting or remineralization of the early caries lesion (*n* = 37,525). We combined remineralization and fluoride treatments because both approaches constitute a non-restorative (i.e., non-surgical) treatment approach.

For each of the 103,232 classified diagnostic codes, all co-occurring treatment codes at the time of their index visit were also identified and grouped into three categories: enamel (F80, F81), dentin (F82, F83, F84), or sealant (F892, F893). These were used to adjust for the overall medical complexity of the patient’s mouth in multivariate analysis.

## Results

Table 2 presents estimated odds ratios from the baseline regression models of the likelihood of restoration that includes patient-level covariates and year of diagnosis. For the first model, year-specific fixed-effects were found preferable to a secular trend (*p*-value of LR test < .001). Patient sex (*n* = 23,224), race (*n* = 32,224), and ethnicity (*n* = 32,356) were not recorded for a significant portion of the sample and could not be used as control variables. The final baseline model includes patient-level covariates for year of diagnosis, patient age, and co-occurring findings. No provider factors were found significant at the 10% level and were not retained (see Additional file 1: Table S1). For the second model that focused upon providers who did not attend the 2008 meeting, the same patient factors as the first, along with a provider-level random effect, were included in the final specification. In addition, the provider factors of total years practicing (*p* = .02) and total years at HPDG were significant (*p* = .04) and retained.

**Table 2:** **Impact of patient demographics upon likelihood of restoration**

	**Including all providers**				**Non-attendees only**	
	**Odd ratio**	**95% CI**	**Odd ratio**	**95% CI**	**Odd ratio**	**95% CI**
Study year						
2005	1	ref	1	ref	1	ref
2006	0.986	(0.920, 1.056)	0.985	(0.920, 1.055)	0.945	(0.869, 1.029)
2007	0.810	(0.758, 0.865)	0.810	(0.758, 0.864)	0.758	(0.699, 0.823)
2008	0.730	(0.685, 0.777)	0.729	(0.685, 0.777)	0.842	(0.763, 0.930)
2009	0.414	(0.390, 0.440)	0.414	(0.390, 0.440)	0.512	(0.452, 0.581)
2010	0.270	(0.255, 0.287)	0.270	(0.255, 0.287)	0.316	(0.279, 0.358)
2011	0.219	(0.207, 0.232)	0.219	(0.206, 0.232)	0.238	(0.210, 0.270)
Patient-level effects						
Age						
<18	0.798	(0.770, 0.827)	0.798	(0.770, 0.827)	0.811	(0.775, 0.849)
18–39	1	ref				
40–50	1.360	(1.298, 1.424)	0.359	(1.298, 1.424)	1.419	(1.337, 1.506)
51–64	1.698	(1.614, 1.787)	1.697	(1.613, 1.786)	1.824	(1.705, 1.952)
65–84	1.656	(1.543, 1.776)	1.656	(1.544, 1.776)	1.742	(1.586, 1.914)
85^a^	1.332	(1.158, 1.532)	1.332	(1.158, 1.531)	1.249	(1.050, 1.486)
Co-occuring findings^a^						
Number of other enamel diagnostic codes	3.942	(3.929, 3.956)	1.011	(1.007, 1.014)	1.012	(1.008, 1.017)
Number of other dentin diagnostic codes	2.940	(2.854, 3.028)	1.050	(1.019, 1.082)	1.046	(1.008, 1.086)
Repaired sealants with damage	5.516	(5.251, 5.795)	1.533	(1.459, 1.610)	1.382	(1.289, 1.482)
Provider-level effects						
Male			1.109	(0.709, 1.737)	1.024	(0.633, 1.659)
Years since graduation			0.990	(0.952, 1.030)	0.990	(0.953, 1.028)
Years in dental group			1.011	(0.967, 1.056)	1.026	(0.985, 1.069)
Provider DPBRN engagement^b^						
Surveys only					0.760	(0.394, 1.467)
Surveys and Studies					0.455	(0.259, 0.800)
Surveys, Studies, and Meetings					0.783	(0.446, 1.375)
Goodness of fit						
Log likelihood	−62,398.810		−62,387.780		−39,101.240	
Aikaike information criterion	124,829.600		124,813.600		78,248.490	
Bayesian information criterion	124,983.000		124,995.700		78,458.610	
Deviance	124,797.600		124,775.600		78,202.490	

Estimates from logistic regression with random provider effects.

^a^Estimated odd ratio compares no findings to average number among those with any findings.

^b^Relative to no DPBRN engagement.

### Research question 1: are the practice patterns of providers (dentists) who elect to participate in a PBRN in greater concordance with current evidence than those who do not participate?

Table 3 contains unadjusted and adjusted (by factors listed in Table 2) restoration rates by level of PBRN engagement. The first column, which corresponds to 2005 restoration rates, indicates after adjusting for patient-level factors, providers who chose no PBRN involvement restored at a significantly higher rate (93%; 95% CI = 92.4%, 93.7%) than other providers with some level of PBRN engagement. The five providers with the highest level of PBRN engagement restored at the lowest rate, 79.5% (95% CI = 78.6%, 80.4%); this rate was significantly lower than the rate among providers who only responded to PBRN surveys, but this was not significantly different from other, higher levels of PBRN engagement.

**Table 3:** **Unadjusted and adjusted restoration rates by PBRN engagement level**

	**2005**	**2006**	**2007**	**2008**	**2009**	**2010**	**2011**
Unadjusted							
No involvement (N = 6)	88.2%	81.5%	75.9%	81.5%	66.9%	56.1%	49.0%
95% CI	(86.6%, 89.9%)	(79.9%, 83.0%)	(74.2%, 77.6%)	(79.9%, 83.0%)	(65.3%, 68.5%)	(54.5%, 57.7%)	(47.5%, 50.5%)
Surveys only (*N* = 4)	85.2%	79.1%	73.5%	77.5%	71.9%	64.7%	59.1%
95% CI	(83.1%, 87.3%)	(76.7%, 81.4%)	(70.9%, 76.1%)	(75.1%, 79.9%)	(69.2%, 74.6%)	(62.2%, 67.2%)	(56.6%, 61.7%)
Surveys and Studies (*N* = 11)	76.8%	78.8%	76.9%	75.1%	59.8%	48.3%	39.7%
95% CI	(75.3%, 78.4%)	(77.3%, 80.3%)	(75.4%, 78.3%)	(73.7%, 76.4%)	(58.4%, 61.2%)	(47.0%, 49.6%)	(38.5%, 41.0%)
Surveys, Studies, and Meetings (*N* = 9)	79.0%	78.9%	78.0%	73.8%	65.7%	57.8%	53.5%
95% CI	(77.5%, 80.5%)	(77.4%, 80.4%)	(76.6%, 79.3%)	(72.5%, 75.0%)	(64.3%, 67.0%)	(56.5%, 59.0%)	(52.2%, 54.7%)
Surveys, Studies, Meetings, and Presentations (*N* = 5)	73.0%	78.4%	74.3%	63.1%	51.1%	42.8%	45.6%
95% CI	(71.0%, 75.0%)	(76.5%, 80.3%)	(72.4%, 76.1%)	(61.1%, 65.2%)	(49.2%, 53.0%)	(41.3%, 44.4%)	(44.1%, 47.0%)
Adjusted^a^							
No involvement (*N* = 6)	93.0%	89.0%	84.2%	87.6%	76.1%	66.5%	59.3%
95% CI	(92.4%, 93.7%)	(88.4%, 89.6%)	(83.5%, 85.0%)	(86.9%, 88.3%)	(75.4%, 76.9%)	(65.7%, 67.2%)	(58.6%, 60.1%)
Surveys only (*N* = 4)	86.6%	81.3%	76.1%	80.8%	75.8%	64.8%	55.4%
95% CI	(85.6%, 87.7%)	(80.1%, 82.4%)	(74.8%, 77.4%)	(79.7%, 82.0%)	(74.5%, 77.1%)	(63.5%, 66.1%)	(54.0%, 56.7%)
Surveys and Studies (*N* = 11)	80.6%	81.7%	79.9%	78.5%	64.5%	52.2%	43.3%
95% CI	(79.9%, 81.3%)	(81.0%, 82.4%)	(79.1%, 80.6%)	(77.8%, 79.1%)	(63.8%, 65.2%)	(51.6%, 52.9%)	(42.6%, 43.9%)
Surveys, Studies, and Meetings (*N* = 9)	82.5%	83.6%	82.1%	78.7%	71.8%	63.8%	60.5%
95% CI	(81.8%, 83.3%)	(82.9%, 84.2%)	(81.5%, 82.8%)	(78.1%, 79.3%)	(71.2%, 72.4%)	(63.2%, 64.5%)	(59.8%, 61.1%)
Surveys, Studies, Meetings, and Presentations (*N* = 5)	79.5%	83.3%	80.8%	72.3%	59.2%	52.3%	54.0%
95% CI	(78.6%, 80.4%)	(82.5%, 84.2%)	(79.9%, 81.6%)	(71.3%, 73.2%)	(58.2%, 60.1%)	(51.5%, 53.1%)	(53.2%, 54.7%)

^a^Estimated from multivariated logistic regression with random provider effects and demographic factors (Table 2).

### Research question 2: does provider participation in a PBRN increase concordance of current practice with current evidence?

Annual restoration rates by level of PBRN engagement are listed in Table 3 for the 7 years included in this study. Examination of both unadjusted (upper portion) and adjusted (lower portion) rates indicates four findings of interest. First, among providers with higher levels of PBRN involvement, there was little change in restoration rates during the first three study years (2005–2007). For instance, in 2005, the nine providers in the surveys, studies, and meetings group restored 82.5% (95% CI: 81.8%, 83.3%) of findings, and in 2007, they restored 82.1% (81.5%, 82.8%). Second, during this same time-frame (2005–2007), restoration rates among providers with little or no PBRN involvement trended toward those of providers with greater involvement. In 2005, providers in the surveys only group restored 86.6% (85.6%, 87.7%) of early lesions, and this rate decreased to 76.1% (74.8%, 77.4%) in 2007. Third, from 2007 to 2008, which was the year of the PBRN meeting disseminating findings from the caries study, while there were *significant increases* in restoration rates within the two groups with little or no PBRN engagement, there were *significant reductions* in restorations rates among the three groups of providers with PBRN engagement with greater engagement leading to greater drops in restoration rates. Fourth, during the last 3 years of the study (2009–2011), restoration rates across all groups decreased significantly. These findings are illustrated in Figure 1 which plots annual restoration rates for three of the five groups (No Involvement, Surveys and Studies, and Surveys, Studies, Meetings, and Presentations). At baseline (2005), the No Involvement group of providers restored early caries lesions at a significantly higher rate; however, their care pattern appeared to converge to the other, more-engaged groups by 2007. In 2008, the most engaged groups (Surveys, Studies, Meetings, and Presentations) decreased their restoration rate by 8.5%, while the other two groups did not begin to significantly decrease restorations until 2009.

**Figure 2:**
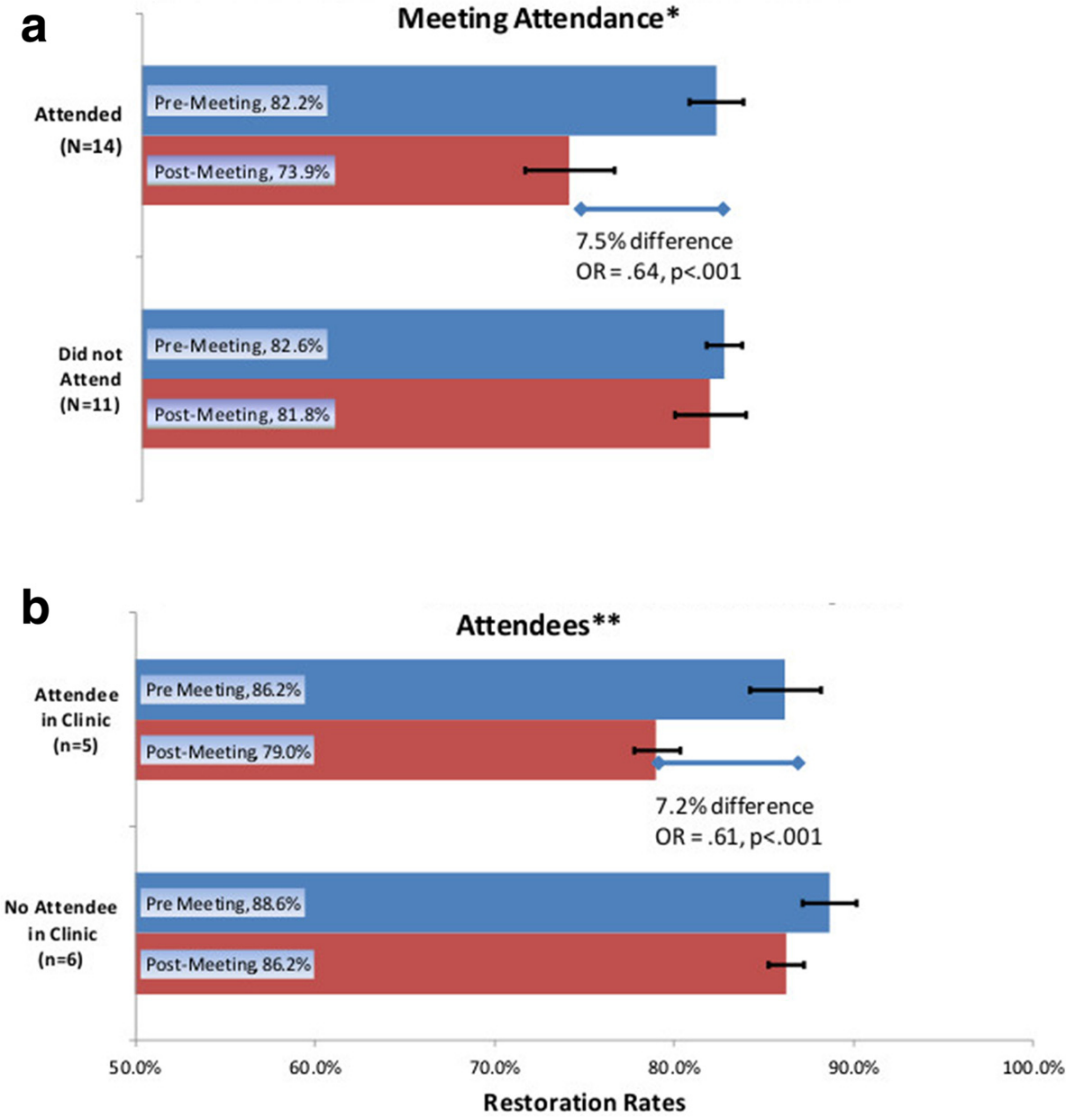
**Clinicians attending network dissemination of meeting and their impact on colleagues. (a)** Change in restoration rates by 2008 National DPBRN meeting attendance*. **(b)** Change in 2008 restoration rates among non-attendees**.

A sharper illustration of the change in care patterns that occurred following the May 2008 PBRN meeting of practitioners is provided in the upper portion of Figure 2, which contrasts restoration rates prior to the meeting with those after the meeting. Among the 35 providers included in the study, 14 attended the 2008 conference (all of those in the 2 most-engaged groups and 10 of the 11 providers in the Surveys and Studies group). Prior to the meeting, attendees restored 82.2% of tooth surfaces with diagnostic codes. After the meeting, attendee restoration rates dropped to 73.9%. In contrast, there was no significant change among non-attendees (pre-meeting: 82.6%; post-meeting: 81.8%).

### Research question 3: do providers who participate in PBRN activities disseminate knowledge to their colleagues?

The lower half of Figure 2 contrasts pre- and post-2008 meeting restoration rates. Contrasts are made between: (1) practitioners who did not attend the 2008 meeting, but who practiced in a HPDG clinic in which at least one other practitioner attended the 2008 meeting and (2) practitioners who did not attend the 2008 meeting, and no other practitioner in the clinic attended the 2008 PBRN meeting. Following the 2008 meeting, providers practicing in a clinic in which there was at least one meeting attendee significantly reduced their restoration rates by 7.2% (86.2% to 79%). In contrast, providers at clinics where no provider attended the 2008 meeting decreased their restoration rates by only 2.4% (88.6% to 86.2%).

## Discussion

Results from this study support the conclusion that dentists who participate in a PBRN are more interested in evidence-based practice, based on the fact that practitioners’ baseline practice pattern for treatment of early caries was more concordant with current evidence [23]. This finding is not surprising because a PBRN is focused on generating new evidence that can improve clinical practice. Dentists with high involvement in the PBRN did change practice, but changes were not significant until the targeted dissemination meeting in 2008 (see Figure 1). This observed practice change using clinical data is consistent with questionnaire-based studies that reached the same conclusion [9,10]. A nearly 10% reduction in restoration of teeth being restored has significant oral health implications when considering the long-term implications to a tooth that may avoid further treatments such as restoration replacement, a crown, or extraction.

A theory base can be very useful in understanding the changes observed [24,25]. Friedson examined physician behavior and concluded that behavior change is derived from three sources [26]. The first is “biography”, such as family and medical training background, which weakly predict behavior. The second is social environment, which strongly influences physician behavior. The third is material self-interest, which physicians were reluctant to admit. Since this study was retrospective, we could not design the study utilizing a theoretical framework to guide measurement of variables associated with observed changes. Implementation science is still in the early stages of its development and we need to better understand the barriers to implementation of the evidence so that interventions can be designed to address them. The PBRN is an organization that connects dentists who share common goals of generating clinically relevant evidence that can improve the care they deliver. The 2008 network-wide meeting was a highly interactive meeting, with breakout sessions that included discussions of clinician concerns regarding patient communication and practicing outside perceived local community norms. Social environment [27] is a factor impacting behavior and these results suggest that the peer influence was strongly associated with changing provider behavior. This and future work would be strengthened by applying an appropriate theoretical framework.

Interestingly, the practice changes that we observed were not limited to meeting participants. We also observed subsequent practice change for dentists practicing in the clinic of attendees. This suggests that meeting attendees had an impact on the peers within the social environment of the clinic where they frequently interact [27]. This is plausible because the HPDG is a large group practice with 17 clinics. Each clinic has 2–5 dentists who share office space and have multiple opportunities to discuss clinical care. HPDG has clinical care guidelines including a caries guideline that existed long before the start of the PBRN (http://www.guideline.gov/content.aspx?id=12538). As stated in the introduction, creation of clinical guidelines alone does not change practice. In order to explore potential mechanisms explaining the effect, we examined the role of the 14 HPDG 2008 PBRN meeting attendees. Many of these individuals had a leadership role, a mentoring role, and/or guideline development role within their practice settings. They were typically viewed by their peers as very competent and respected clinicians. One might surmise that these individuals were effective change agents.

We also know that the HPDG leaders (clinical and administrative) decided to implement a modest financial incentive in 2010 and 2011. It was part of a non-production incentive designed to reinforce clinical goals. This study was not designed to measure the impact of a financial incentive, but potentially, this payment incentive contributed to the changes in practice of non-PBRN dentists and the sustained change of PBRN-enrolled dentists observed in 2010–2011. A review of pay for performance in dentistry suggests that payments aligned with current best evidence defining quality care are effective at changing clinical practice [28]. The practice change we observed in 2010–2011 impacted practitioners who had not changed practice patterns due to PBRN activities. The actual amount of the incentive was modest. Other initiatives in addition to ‘pay for performance’ occurred during this time period. They include the possible impact of opinion leaders and changes to the electronic dental record where a new window was created to systematically collect information related to remineralization. The impact of each of these changes cannot be delineated but needs to be considered in the changes observed.

Dissemination and implementation science seeks to understand how to systematically facilitate utilization of evidence by understanding successful strategies for adoption and sustainability of evidence-based interventions [29]. Results from this study suggest that PBRN engagement may be an effective means to foster movement of the latest evidence into routine clinical practice.

Limitations of this study include the retrospective design and our reliance on data entered into a clinical record. Clinical data in the electronic health record have not been fully validated and may not accurately reflect the actual clinical condition. The issue of missing data is also of concern. While uniformly distributed across all provider groups, missing treatment plan data may have impacted results. As with any study, findings are only as strong as the data that underlie them. In this study, there were missing data across all provider groups. This limited our ability to adjust for demographic factors in multivariate analysis. Further, we only examined the practice patterns of 35 providers; however, we were able to have a large number of observations for each provider. These providers practice in a large staff model setting that may not be representative of other practice settings. For these reasons, we consider these findings supportive of additional investigation but not conclusive on their own. Ideally, we would like to replicate these findings in other settings, but currently, dentistry lacks standardized electronic clinical data that includes universally accepted diagnosis codes. Patient preference was not measured and may have contributed to observed changes. The prior regional Dental PBRN did conduct a study that examined patient satisfaction with a restoration visit and found that patients frequently preferred more information about the restoration choices beyond that provided [30]. A randomized trial of providers is a stronger study design but has several limitations, including feasibility and the inability to identify the components of PBRN participation that are most predictive of practice change.

## Conclusions

PBRNs conduct research in community-based settings to answer clinically relevant questions that seek to improve practice and achieve better patient outcomes. Results from this study are consistent with a growing body of literature that suggests PBRNs are an effective approach not only to conduct studies representing community-based populations but also to disseminate and implement evidence into routine practice if network activities are topic- and treatment-specific, collegial in nature, and highly participatory. The PBRN impact appears most meaningful to engaged providers, but dissemination to colleagues was observed. Further research is needed to understand those mechanisms.

## Additional file

Additional file 1:
**Technical appendix.** The following file contains additional information regarding how the final analytic dataset was defined and the final empirical models developed. It contains three tables, and a brief description of each table is provided. **Table S1.** Description of diagnostic and treatment codes used in the study. **Table S2.** Logistic regression models of restoration probability. **Table S3.** Logistic regression models of restoration probability among non-attendees.
